# Editorial: Complement: Latest developments regarding structure, mechanism, and connections to other proteolytic pathways

**DOI:** 10.3389/fimmu.2023.1144038

**Published:** 2023-01-24

**Authors:** József Dobó, Péter Gál, Brian V. Geisbrecht

**Affiliations:** ^1^ Institute of Enzymology, Research Centre for Natural Sciences, Budapest, Hungary; ^2^ Department of Biochemistry & Molecular Biophysics, Kansas State University, Manhattan, KS, United States

**Keywords:** proprotein convertases, MASP-3, age-related macular degeneration (AMD), C2, heme, Borrelia, gC1qR, coagulation

It is with great pleasure that we introduce you to the Research Topic entitled “Complement: Latest Developments Regarding Structure, Mechanism, and Connections to Other Proteolytic Pathways”. Consisting of two focused review articles, seven original research articles, and one brief report, we think that this collection makes for an attractive cross-section of current basic research endeavors in the topic area. Furthermore, as these contributions arise from a range of approaches including *in vivo* animal studies, single-cell transcriptomics, interaction analysis, structural biology, and assays of immune function, we believe this collection provides an excellent showcase of the diversity of experimentation and thought within the field. With that in mind, the following paragraphs provide brief synopses of the individual manuscripts and the scientific context from which they were written.

As proteins destined for the extracellular environment, the biogenesis of various complement components is necessarily complex. This is even more the case for the numerous complement components that must undergo processing from their respective proproteins to their mature forms. In this review, Dobó et al. discuss the role of proprotein convertases on the intracellular and extracellular processing complement proteins and their overall contributions to complement function. With new roles for complement being reported regularly, this review provides a useful resource for understanding the basic events necessary for generating fully active complement components. These considerations are especially relevant in light of the continually emerging role of the intracellular complement system.

Of course, another layer of regulation must be considered when complement components assemble into multipartite functional units, such as the initiating complexes of the classical (CP) or lectin (LP) pathways. Although MASP-3 has been shown to circulate in the blood in its active form, how this is influenced by its interaction with LP pattern recognition molecules remains unknown. In their manuscript, Kusakari et al. use a series of recombinant forms of MASP-3 to investigate its activation, assembly, and clearance in experimental mice. These authors showed that while MASP-3 binding to pattern recognition molecules is not required for MASP-3 activation, it does appear to influence the kinetics of MASP-3 retention in the blood. Since MASP-3 has been shown previously to activate pro-factor D, these observations may inform future efforts aimed at developing inhibitors of the complement alternative pathway (AP).

When mentioning therapeutic development, the complement system has received considerable attention recently due to the approval of the C3 inhibitor, pegcetacoplan, for treatment of paroxysmal nocturnal hemoglobinuria (PNH). This same compound is currently undergoing evaluation for treatment of age-related macular degeneration (AMD), which is characterized by excessive C3 activation in the retina and surrounding tissues. However, mixed results for other complement inhibitors in AMD trials led Zauhar et al. to investigate complement homeostasis in the eye at single-cell resolution. These authors found temporal and spatial differences in local complement component production between healthy and diseased eyes. Together, their observations provide a more nuanced understanding of complement’s roles in maintaining function of the retina and surrounding tissues, and how it might be inhibited to therapeutic benefit.

Whereas changes in local complement component production in the eye have only recently been discovered, the genetic risk factors linked to development of AMD have been known for some time. Unfortunately, the functional consequences of many rare variants in Factor H (FH) and Factor I (FI) remain unknown. To address this issue systematically, Hallam et al. devised a method for generating recombinant FI and thereafter developed a real-time assay for assessing formation of the ternary complex between C3b, FH, and FI. Interestingly, these authors showed that certain rare variants in FI appear to have dominant negative effects by competing with wild-type FI for binding to the complex of C3b and FH. The work described in this manuscript should permit design of more effective interventions for AMD that account for individuals’ genotypes.

Although FH has been known as the primary regulator of the AP for decades, the functions of a family of FH-related proteins (FHRs) have become apparent more recently. As with FH, coding variants in FHR1 have been linked to the development of atypical hemolytic uremic syndrome (aHUS), which is a rare thrombotic microangiopathy that can lead to end-stage renal disease. To better understand the mechanism through which FHR1 variants may contribute to disease, Xu et al. examined the effects of two isoforms known as FHR1*A and FHR1*B on regulation of the AP. These authors uncovered several functional differences for FHR1*B, including an increased affinity for C3b and a greater ability to impede FH regulatory activities. Together, these observations enhance understanding of FHR function and provide new insights into the molecular events that may underlie aHUS development in certain patients.

As with aHUS patients, those with the rare kidney diseases known as C3 glomerulopathies display evidence of unregulated complement activity. While many such individuals have gain-of-function coding variants in components of the AP, such as Factor B, newer studies have identified patients that harbor mutations in complement component C2. In this brief report, Kuźniewska et al. investigated the functional consequences of patient-derived mutations in the von Willebrand factor A (vWA) domain of complement C2. Their work further establishes the functional analogies between Factor B and C2 and reveals that mutations in the vWA domain of these paralogs may have unpredictable consequences on complement activity.

The synopses above illustrate examples of genetic variations leading to changes in complement component function. However, the possibility that endogenous small molecules might also affect complement components remains relatively unexplored. Since free heme has been described as an activator of the AP, Gerogianni et al. sought to define the mechanism behind its action. These authors showed that heme binds directly to FI and inhibits its ability to degrade soluble and surface-bound C3b in the presence of either FH or soluble complement receptor-1 as a cofactor. Interestingly, they also found that the heme-scavenging protein, hemopexin, elicited a protective function toward FI, so long as it was present prior to exposure to free heme. These observations have important implications for regulation of the AP in hemolytic disorders.

When discussing mechanisms of complement regulation, it is almost impossible to ignore the many lessons learned from studying pathogen-derived immune evasion proteins. In this manuscript, Booth et al. continue their recent line of investigation into the BBK32 family of CP inhibitors produced by *Borrelia* spp. Using biochemical, structural, and functional studies, these authors found that FbpA and FbpB share the ability of *B. burgdorferi* BBK32 to bind C1r and inhibit its activity. Interestingly, they also found that while FbpA binds to both zymogen and activated C1r, FbpB is selective for activated C1r alone. These results not only further understanding of complement evasion by an emerging human pathogen, they provide new information on a family of inhibitors selective for the activation state of complement component C1r.

Given the title of this collection, we think investigating connections between complement and other proteolytic pathways is a promising area of research. Indeed, one of the best understood of these connections is the interaction between gC1qR (a potent initiator of the CP), coagulation factor FXII, high-molecular weight kininogen (HMWK), and prekallikrein (PK) that occurs on the surface of endothelial cells in response to inflammatory stimuli. In this manuscript, Zhang et al. use a structure/function approach to further define the gC1qR/FXII interaction and explore how it is perturbed by a panel of monoclonal antibodies. Since the gC1qR/FXII/HMWK/PK complex triggers excessive generation of the vasodilator bradykinin in angioedema, these results provide new insights into how assembly of this complex might be inhibited as a potential treatment.

The current emphasis on therapeutic discovery is likely to continue for the foreseeable future, yet is best positioned when it seeks to manipulate events whose functional consequences are well established. Clearly, much remains to be discovered. To that end, Pryzdial et al. conclude this collection with a review article that examines the many biochemical and cellular connections between complement and coagulation. By changing our focus from one that looks at individual pathways to a more wholistic view that treats these two proteolytic systems as coresident in the same physiological compartment, the authors seek to remove many of the distinctions made for convenience at the expense of understanding. In doing so, they set the stage for identifying new points of crosstalk between complement, coagulation, and still other proteolytic pathways.

We hope you find this collection timely, informative, and thought-provoking for new lines of investigation into the complement system and other proteolytic pathways.

## Author contributions

BG drafted the editorial manuscript. JD and PG corrected the manuscript. All authors approved its final version.

